# Reply to: The Perme scale score as a predictor of functional status and complications after discharge from the intensive care unit in patients undergoing liver transplantation

**DOI:** 10.5935/0103-507X.20190083

**Published:** 2019

**Authors:** Camila Santos Pereira, Alexandra Torres de Carvalho, Adriane Dal Bosco, Luiz Alberto Forgiarini Júnior

**Affiliations:** 1 Centro Universitário Metodista IPA - Porto Alegre (RS), Brasil.; 2 Complexo Hospitalar Irmandade Santa Casa de Misericórdia de Porto Alegre - Porto Alegre (RS), Brasil.; 3 Curso de Fisioterapia e Programa de Pós-Graduação em Saúde e Desenvolvimento Humano, Universidade La Salle - Canoas (RS), Brasil.

**To the Editor**

First, the authors are thankful for the comments and for the opportunity to clarify points of our study published in Revista Brasileira de Terapia Intensiva.^(1)^ Our research line has involved investigating some functional assessment tools in different populations to identify functional behavior. In the study referred to above, we evaluated the use of the Perme Mobility Score in the intensive care unit during the postoperative period of liver transplantation.

We agree with the observation regarding the time spent on the transplant waiting list and its functional impact on these patients, as demonstrated by Casales et al.,^(2)^ and note that this variable is also a marker of mortality according to Galant et al.^(3)^ We are currently working on a future study in which the time spent on the transplant waiting list will be correlated with the functional assessment score to determine its possible impact and/or relationship. Regarding the issue of the nonexclusion of patients diagnosed with hepatopulmonary syndrome, we believe that during the posttransplant period, this variable (dyspnea) does not have an important impact on the functional assessment.

Regarding the use of the Perme score outside the intensive care unit, we agree with the observations made. In fact, our intention was to allow comparability by using the same tool, despite knowing that we could have used the Functional Independence Measure (FIM) or the Barthel index, as we have previously done when assessing critically ill patients after discharge from the ICU.^(4)^ An important point in this case would be to use a specific tool at each time point and to evaluate and compare their results through common points measured with the International Classification of Functioning (ICF).

We are thankful for the constructive observations, which, in our opinion, tend to raise the quality of future studies and foster scientific discussion.

## References

[1] Pereira CS, Carvalho AT, Bosco AD, Forgiarini Júnior LA (2019). Escala Perme como preditor de funcionalidade e complicações após a alta da unidade de terapia intensiva em pacientes submetidos a transplante hepático. Rev Bras Ter Intensiva.

[2] Casales da Silva Vieira R, Álvares-da-Silva MR, de Oliveira AR, da Silveira Gross JS, Kruger RL, Dal Bosco A (2018). Cirrhosis affects maximal oxygen consumption, functional capacity, quality of life in patients with hepatitis C. Physiother Res Int.

[3] Galant LH, Forgiarini Junior LA, Dias AS, Marroni CA (2013). Maximum Oxygen Consumption Predicts Mortality in Patients with Alcoholic Cirrhosis. Hepatogastroenterology.

[4] Curzel J, Forgiarini Junior LA, Rieder MM (2013). Avaliação da independência funcional após alta da unidade de terapia intensiva. Rev Bras Ter Intensiva.

